# UVA-Triggered Drug Release and Photo-Protection of Skin

**DOI:** 10.3389/fcell.2021.598717

**Published:** 2021-02-11

**Authors:** Vega Widya Karisma, Wei Wu, Mingxing Lei, Huawen Liu, Muhammad Farrukh Nisar, Matthew D. Lloyd, Charareh Pourzand, Julia Li Zhong

**Affiliations:** ^1^Key Laboratory of Biorheological Science and Technology, Ministry of Education, Bioengineering College, Chongqing University, Chongqing, China; ^2^Three Gorges Central Hospital, Chongqing, China; ^3^Department of Physiology and Biochemistry, Cholistan University of Veterinary and Animal Sciences (CUVAS), Bahawalpur, Pakistan; ^4^Drug and Target Discovery, Department of Pharmacy and Pharmacology, University of Bath, Bath, United Kingdom; ^5^Medicines Design, Department of Pharmacy and Pharmacology, University of Bath, Bath, United Kingdom; ^6^Medicines Development, Centre for Therapeutic Innovation, University of Bath, Bath, United Kingdom

**Keywords:** caged iron chelators, drug delivery systems (DDS), skin photo-protection, smart sunscreens, up-conversion nanoparticles, UVA-triggered drug release

## Abstract

Light has attracted special attention as a stimulus for triggered drug delivery systems (DDS) due to its intrinsic features of being spatially and temporally tunable. Ultraviolet A (UVA) radiation has recently been used as a source of external light stimuli to control the release of drugs using a “switch on- switch off” procedure. This review discusses the promising potential of UVA radiation as the light source of choice for photo-controlled drug release from a range of photo-responsive and photolabile nanostructures *via* photo-isomerization, photo-cleavage, photo-crosslinking, and photo-induced rearrangement. In addition to its clinical use, we will also provide here an overview of the recent UVA-responsive drug release approaches that are developed for phototherapy and skin photoprotection.

## Introduction

The introduction of targeted drug delivery systems (DDS) has greatly enhanced the bioavailability of the drugs while substantially reducing their side effects. This is because DDS enable the highly selective delivery of the drug to the lesion site whilst minimizing its premature loss (Bahrami et al., 2017; Breitsamer and Winter, 2019; Huang et al., 2019). It also provides flexibility by controlling the timing, duration and the level of drug release while combining the attainment of a therapeutic drug concentration with on-demand release (Davoodi et al., 2018). The ability to control drug delivery has become a very important goal during drug development since it maximizes therapeutic efficiency whilst minimizing undesirable effects. This is achieved with either internal or external stimuli (Bhowmik et al., 2012; Senapati et al., 2018). The use of DDS responding to external stimuli offers a promising approach for the controlled release of drugs since their spatiotemporal properties allow flexible control of drug dosage, timing, and duration of treatment (Yao et al., 2016; Wang and Kohane, 2017; Rwei et al., 2018) according to the patients' needs, with consequent reduction in side-effects and an increase in therapeutic efficacy.

It is becoming possible to use either UV or near-infrared (NIR) radiation as an external stimuli in DDS. Light can be used either as a single-dose or a multi-switchable stimulus. The single- light dose stimulus can trigger an irreversible change in the photo-responsive drug carrier which results in the release of the drug. The multi-switchable stimulus allows the drug to be released from its carrier in a pulsatile manner (Alvarez-Lorenzo et al., 2009). Light as a stimulus has been the subject of special attention because it allows release of a wide range of therapeutic compounds with spatial and temporal control (Bagheri et al., 2016). By selecting an appropriate wavelength, it may be possible to accurately fine-tune the power and focus of the light, depending on the energy it bears and its ability to penetrate the body tissues (Barhoumi and Kohane, 2015). The nanostructure-based DDS may open or close upon irradiation (Liu et al., 2016; Shim et al., 2017). In fact, light is an attractive trigger for drug delivery due to its ability to facilitate spatiotemporal drug release using photo-degradation, photo-thermal effects, or photo-induced hydrophobicity transfer (Yu et al., 2014; Son et al., 2019). The presence of light can induce irreversible or reversible structure changes through bond cleavage, isomerization, switchable electrostatic attraction and molecular rearrangement (Kamaly et al., 2016). Activation can occur by a one or two-photon absorption processes (Liu G. et al., 2013).

Ultraviolet A (UVA, 320–400 nm) comprises more than 95% of the UV radiation reaching earth and is responsible for oxidative damage to the skin and eyes. This damage results in premature photo-aging, and contributes to skin carcinogenesis, ocular pathologies and affects the balance between cellular repair and apoptosis (Svobodova et al., 2006; Amaro-Ortiz et al., 2014; Kim and He, 2014). As the longest wavelength of UV radiation, UVA can penetrate to the dermis and subcutaneous layer and 1% of UVA can penetrate to the blood (up to ~150 μm in depth) and can cause oxidative damage to cellular constituents notably DNA, proteins and lipids due to its ability to generate reactive oxygen species (ROS) *via* intracellular chromophores (Seité et al., 2010; D'Orazio et al., 2013; Karran and Brem, 2016; Brem et al., 2017; Lan et al., 2019; Moreno et al., 2019), which may lead to the cell death (apoptosis or necrosis depending on the severity of the insult) (Pourzand and Tyrrell, 1999; Marionnet et al., 2011; Jaszewska et al., 2013; Burke, 2018; Dunaway et al., 2018). UVA irradiation also directly accelerates skin aging by promoting the formation of wrinkles, reducing elasticity and loss of skin pigmentation due to upregulation of collagen levels and matrix metalloproteinases (MMPs) (Seité et al., 2010; Marionnet et al., 2011; Dunaway et al., 2018).

Despite the adverse effects of solar UV radiation, its energy especially UVB is absorbed by the epidermis layer through 7-dehydrocholesterol to convert the energy photon into provitamin D_3_ and isomerized into vitamin D_3_ to regulate the Ca^2+^ metabolism of the skin (Wexler et al., 2005; Passeron et al., 2019). In dermatology, UVA is routinely used for photo-therapy in combination with topical treatment due to its low side effects and ease of use for many skin conditions (Vangipuram and Feldman, 2016). Nevertheless, UVA irradiation may contribute to skin cancer (Wexler et al., 2005). Long wave UVA1 (340–400 nm) has an effect on localized atopic dermatitis and scleroderma, and exerts its therapeutic effect through T cell apoptosis, collagenase induction, and angiogenesis (Lee et al., 2013; Teske and Jacobe, 2016). The UVA therapy and its combination with psoralen also known as PUVA (Psoralen-UVA) is currently used for the treatment of diseases such as psoriasis, vitiligo, and cutaneous T-cell lymphoma (Tippisetty et al., 2013; Tsai-Turton, 2014). PUVA triggers the delayed reaction of erythemal ~96 h after UVA radiation of the psoralen-sensitized skin (Ibbotson, 2018). The antiproliferative activity of the treatment occurs via the arrangement of DNA monoadduct and inter-strand cross-links (ICLs) in the irradiated cells (Derheimer et al., 2009).

**Figure 1:**
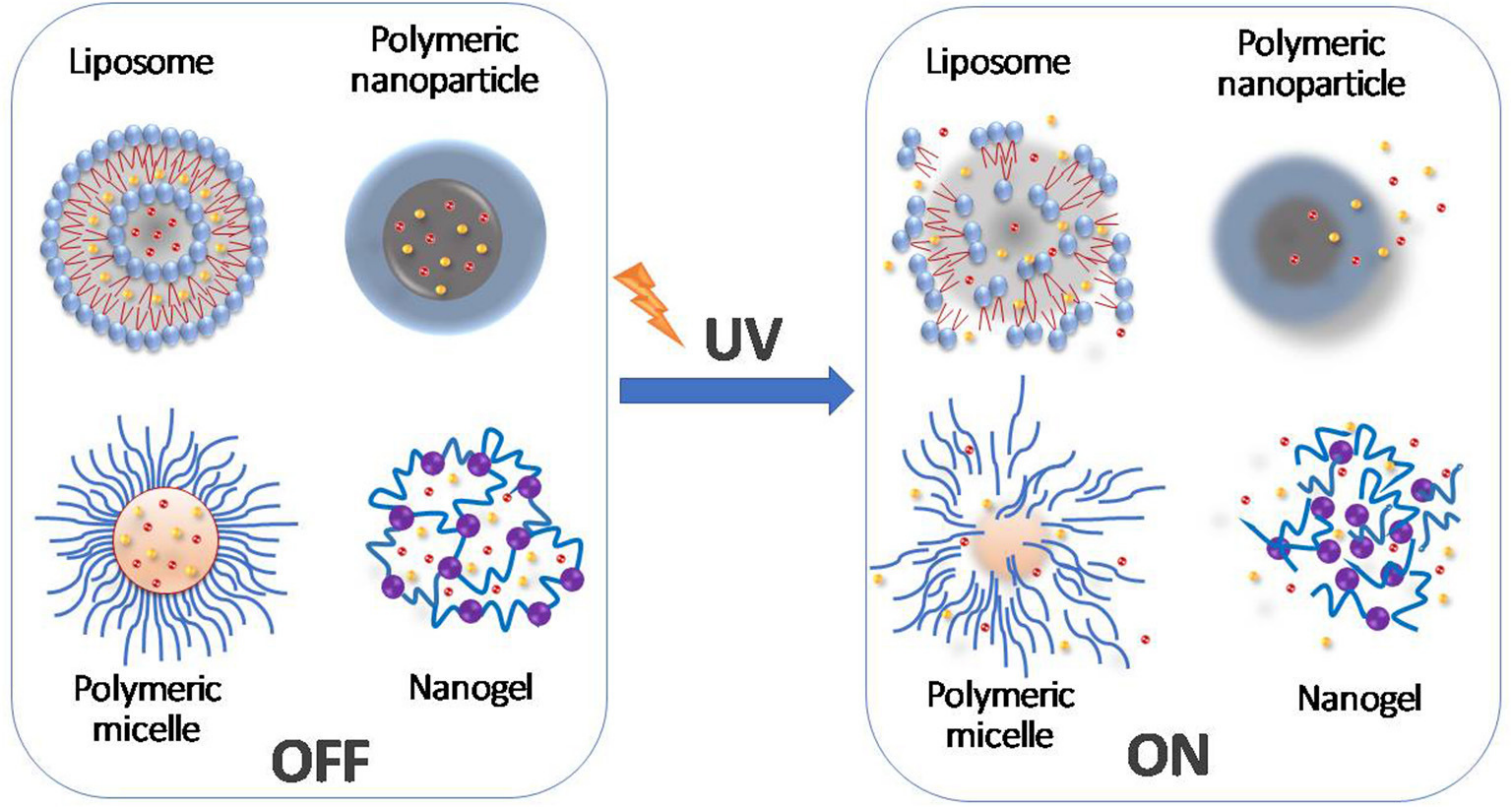
Schematic diagram representing the controlled drug release mediated by the UV light-induced changes to molecular structures.

As an attractive light stimulus, the controlled spatiotemporal use of UVA radiation offers the possibility of activating nanostructure-based DDS following irradiation and the release of the medicine at a specific side (Breuckmann et al., 2004; Jiang et al., 2005; Agasti et al., 2009; Jin et al., 2011; Prasad et al., 2020) (see Figure 1). Some photo-activated substances and functional groups are relatively inert to NIR light but react upon irradiation with UV light, thereby causing chemical and/or physical changes (Jayakumar et al., 2012; Tong et al., 2012). Among several light triggers, UV light has been considered for the controlled release of drugs, proteins, and DNA as well as for other applications in signaling agents (Sreejivungsa et al., 2016).

The UV-responsive drug-carrying nanoparticles allow the release of their cargo following UV radiation *via* various mechanisms of UV-triggered drug delivery systems (Tong et al., 2012; Mura et al., 2013; Barhoumi et al., 2015). Most of the light-responsive materials respond to UV light, leading to significant release of their cargo to targeted site (Zhao et al., 2019). Nanoparticles are capable of discharging their payload at the higher range and farther diffusion through the corneal tissue and collagen gel upon the irradiation with UV light (Tong et al., 2012). The UV light also provides high energy per photon to break the covalent bonds, thus initiating drug-release by photo-isomerization, photo-cleavage, photo-crosslinking or rearrangement (Linsley and Wu, 2017; Zhao et al., 2019). These advantageous matters are considered to utilize UV light in controlling drug delivery. Nevertheless, the high phototoxicity and shorter tissue penetration (up to 100–150 μm) limit the utilization of UV light resulting in tissue damage and low drug release from the DDS (Liu et al., 2016; Zhao et al., 2019). In this regard, a strategy has been established to overcome the challenge of UV light limitation by the use of up-conversion nanoparticles (UCNPs). The use of a combination of NIR and UV to produce UCNPs is an attractive alternative as it minimizes the adverse effects of UV penetration since UCNPs can convert NIR light into UV and visible light (Cho et al., 2015).

Various studies investigating UV-induced drug release are getting considerable attention. For example, Cabane et al. (2011) studied the potential of photo-responsive polymersomes using a photo-cleavable amphiphilic block copolymer (PMLC-2-nitrobenzyl-polyacrylic acid), which degraded upon exposure to UV radiation (365 nm) in a dose-dependent manner. Similarly, Wang et al. (2014) designed micelles incorporating spiropyran within the polymer. Upon irradiation the micelle diameter decreased with consequent release of the drug. A photo-responsive liposome based on 1,2-bis(10,12-tricosadiynoyl)-sn-glycero-3-phosphocholine (DC-8,9-PC) fabricated by Li et al. (2015) targeted to CD20 underwent intermolecular cross-linking *via* its di-acetylenic groups upon UV irradiation. Wang et al. (2015) combined mesoporous titanium dioxide (TiO_2_) with polyethyleneimine (PEI) to produce nanoparticles, which released folic acid and paclitaxel upon UV irradiation thereby facilitating controlled release of the anticancer drug and promoting cancer cell-specific uptake.

UVA-controlled release can also be a promising alternative strategy for protecting the skin from the effects of UV radiation (Vogt et al., 2016). Previous research by us demonstrated that the UVA-controlled release of caged iron chelators can protect the skin against UVA-induced oxidative damage and the ensuing necrotic cell death (Yiakouvaki et al., 2006). This is due to the ability of the caged iron chelators to sequester excess harmful labile iron release in skin cells caused by radiation in a dose- and context-dependent manner (Zhong et al., 2004; Kitazawa et al., 2006). The UVA-mediated labile iron release can be mitigated by the use of an iron chelator thus reducing the associated photo-aging and photo-carcinogenesis (Pouillot et al., 2013) using photo-controlled and activatable-caged iron chelators (Reelfs et al., 2010). Our approach provided the possibility of formulating sunscreens with light-activated iron chelators as their main UVA-activatable ingredient to protect the skin against the oxidizing UVA component of sunlight.

This review highlights the mechanisms underlying drug release following photoactivation of various drug-loaded nanoparticles with UV (specifically UVA) and NIR for clinical use or skin photoprotection.

## Mechanisms of UVA-Induced Drug Release

**Figure 2:**
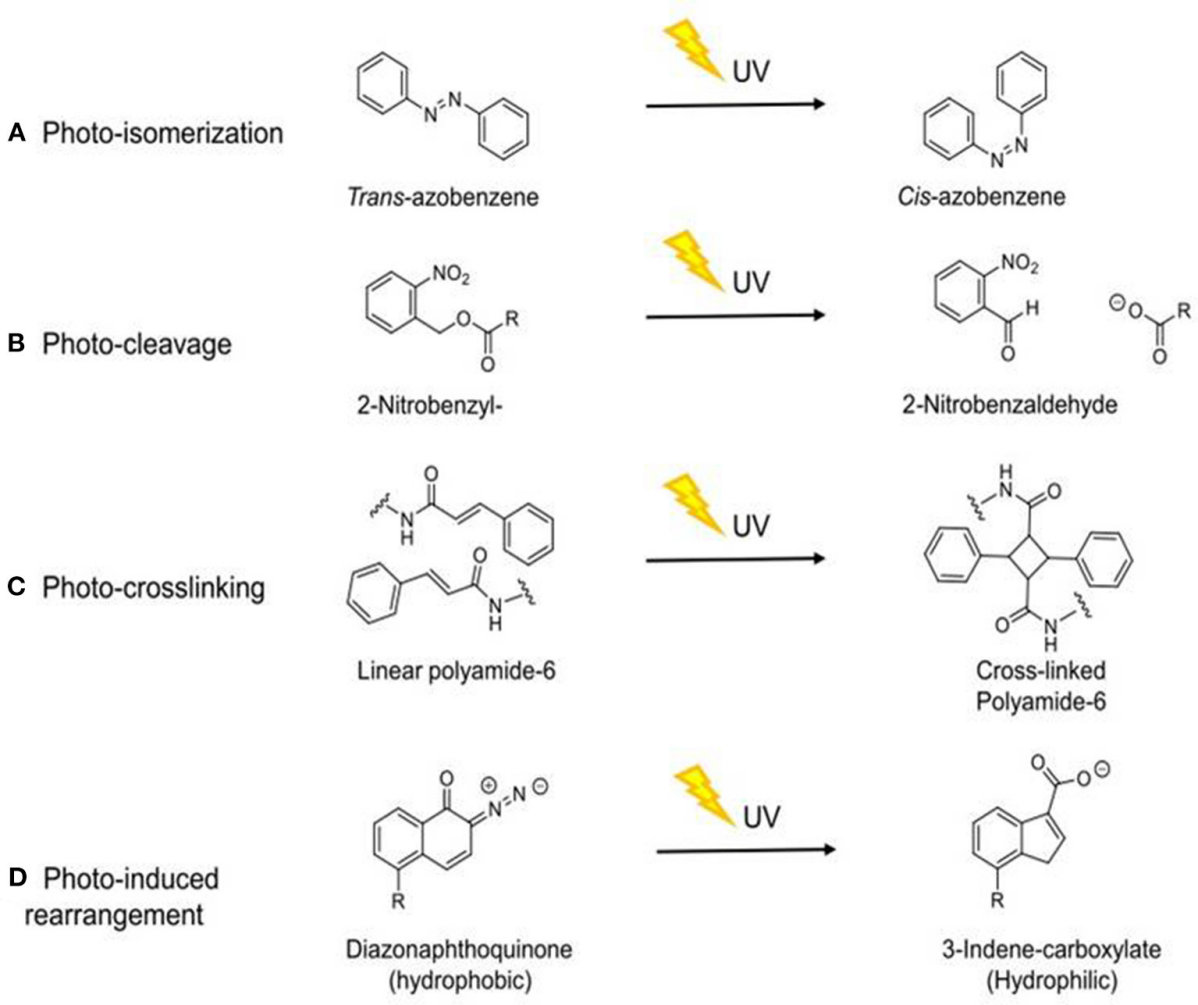
Schematic diagram of the molecular mechanisms by which UVA induces drug release through different mechanisms. **(A)** Photo-isomerization (Stranius and Börjesson, 2017); **(B)** Photo-cleavage (Kauscher et al., 2019); **(C)** Photo-crosslinking (Tunc et al., 2014); **(D)** Photo-induced rearrangement (Olejniczak et al., 2015). *R* = aliphatic group.

In order to use UVA light as a trigger for DDS, specific molecular mechanisms are required. Controlled drug release can be achieved through a number of different mechanisms, including photo-isomerization, photo-cleavage, photo-crosslinking, and photo-induced rearrangement (Barhoumi et al., 2015), as summarized in Figure 2 and discussed below.

### Photo-Isomerization

Photo-isomerization is the interconversion of two metastable forms upon light irradiation, which results in a conformational change due to the two forms having different physical and chemical properties (Stranius and Börjesson, 2017). This photo-isomerization can be reversible (Li et al., 2016). Some isomerization mechanisms involve the breaking of the chemical structure and its conversion into the lowest energy phase following photo-isomerization. These changes can be reversible as seen with spiropyran (Olejniczak et al., 2015). Other molecules such as azobenzene can undergo a reversible *cis*/*trans* isomerization (Shao et al., 2008) without bond breakage (Barhoumi et al., 2015).

The photo-isomerizations of azobenzene and their derivatives have been extensively studied for their use as photochromic compounds in drug-release systems (Gao et al., 2014). Azobenzene can exist as both *cis* and *trans* (*Z*-and *E*-) isomers. The *trans* to *cis* isomerization occurs upon irradiation with UV whilst the reverse *cis*- to *trans*-isomerization is catalyzed by visible light, mechanical stress or electrostatic stimulation (Bandara and Burdette, 2012; Zhang et al., 2019). Compared to many photo-responsive moieties, azobenzene has better light-responsive properties by virtue of its ability to undergo reversible isomerization between the non-polar *trans* and polar *cis* configurations in many solvents (Zhang et al., 2019). Consequently, it has been widely used in liposomes, mesoporous silica, and micellar DDS (Olejniczak et al., 2015). Azobenzene absorbs at two wavelengths, in visible light as a weak band (~430 nm in *n*-hexane) associated with the excitation of the formally dipole-forbidden ^1^B_2g_(nπ^*^) transition, and as a strong band in the UV region (~320 nm in *n*-hexane) linked to absorption of dipole-allowed ^1^B_1u_(ππ*) transition (Tan et al., 2015). Therefore, the isomerization from *trans* to *cis* geometry occurs upon irradiation with UV light (320–365 nm) and is reversed by exposure to blue light (400–450 nm) or heat, thus changing molecule geometry, polarity, and electronic features (Tylkowski et al., 2017).

A study from Galante et al. (2019) investigated bridging of silsesquioxane with azobenzene moieties in order to produce a self-healing material upon UV-irradiation. They demonstrated that UV irradiation effectively changed the intra-molecular conformation of the azo-chromophores resulting from a *trans* to *cis* isomerization. UV irradiation increased the mobility of the material allowing it follow into damaged areas which was followed by reestablishment of hydrogen bonds. Another study from Zhang P. et al. (2019) prepared shape-memory materials (SMMs) incorporating azobenzene derivative 4-cyano-4′-pentyloxyazobenzene (5CAZ) and UCNPs. These mechanically stretched materials could be deformed by the UV/vis/NIR light irradiation due to the presence of the UCNPs. Irradiation of nanoparticles with NIR light (980 nm) causes the emission of photons within the UV/vis region and their absorption by photo-responsive azobenzene derivative resulting in photo-isomerization and hence changes in the UCNP structure (Liu J. et al., 2013).

**Figure 3:**
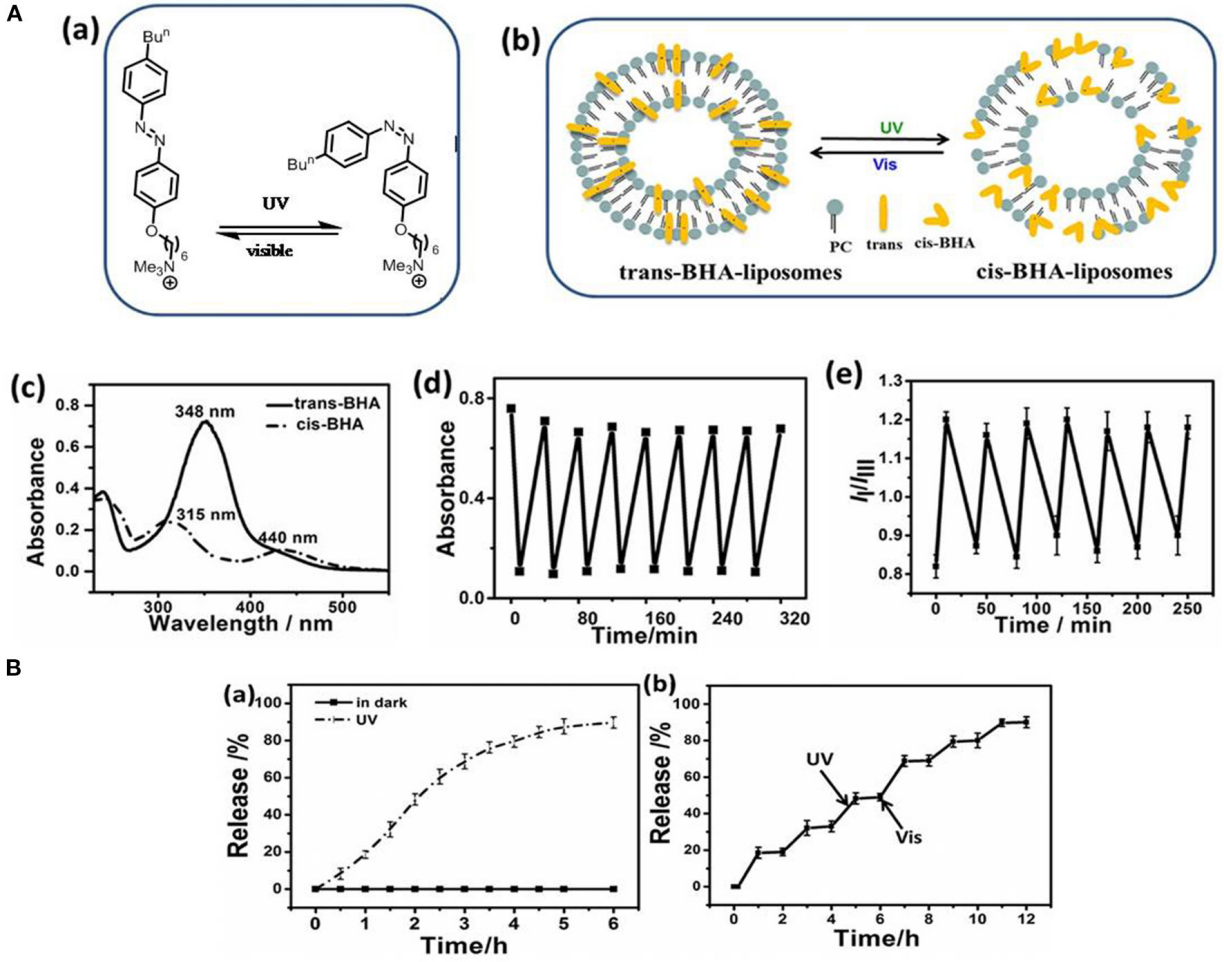
**(A)** Photo-isomerization of BHA-liposome (a) Chemical structure and photo-isomerization of BHA; (b) The microstructure schematic of the trans- and *cis*-BHA-liposome; (c) The UV-visible spectra of BHA-liposomes showing changes upon isomerization; (d) Changes in the absorbance of the BHA-liposome at 348 nm during alternating cycles of UV and visible light; (e) I_*I*_I_*m*_ values for the BHA-liposome with time upon alternating irradiation with UV and visible light. **(B)** Drug release from a liposome: (a) Curcumin release from liposomes in the dark (black line) and upon UV irradiation (red line); (b) The accumulated release of curcumin from BHA-curcumin-liposomes upon alternate irradiation by UV and visible light. Reproduced with permission (Liu and An, 2019). Copyright 2018, Elsevier. BHA, 4-butylazobenzene-4-hexyloxy-trimethyl-ammonium trifluoroacetate.

Liposomes have also been established as an effective light-responsive drug delivery system, as irradiation can induce bilayer isomerization leading to structural changes (Lajunen et al., 2016; Deng et al., 2018). Liu and An (2019) enhanced the photo-responsiveness of liposomes by preparing liposomes that incorporated 4-butylazobenzene-4-hexyloxy-trimethyl-ammoniumtrifluoroacetate (BHA) as a photo-responsive element. Reversible structural isomerization was used to release the entrapped curcumin payload upon irradiation (Figure 3). Similarly, Uda et al. (2016) investigated the release of molecules from liposomes in response to light. The copolymer of poly(vinyl alcohol) carrying a malachite green moiety (PVA-MG) was inert under dark condition but associated with liposomes upon irradiation, causing the release of the encapsulated compound.

### Photo-Cleavage

The implementation of photosensitive drug release through a photo-cleavable linker can be achieved using coumarin or 2*-*nitrobenzyl-derivatives, dendrimersomes or other known cleavable units (Kauscher et al., 2019). Photo-cleavage occurs due to the breakdown of covalent bonds following light irradiation (between 320 and 400 nm), effecting fast degradation of molecule structures and allowing the release of payloads or the release an immobilized molecule (Huang et al., 2014; Grim et al., 2015; Olejniczak et al., 2015). Absorbance of high energy photons by the pro-drug are required to induce photo-cleavage of bonds (Härtner et al., 2007). 2-Nitrobenzyl and coumarin moieties are photo-cleavable linkers commonly incorporated into polymers, micelles, or in low molecular compounds (Härtner et al., 2007) used to construct the drug delivery systems. Photo-cleavage can be achieved either by UV radiation (between 320 and 400 nm) or NIR light (Liu G. et al., 2013).

Kim and Diamond (2006) investigated 2*-*nitrobenzyl and 2*-*nitrophenylethyl derivatives experiencing photo-decomposition upon irradiation with long-wavelength UV light (UVA, 365 nm) in DDS. These studies revealed that the rate of decomposition for 2*-*nitrobenzylderivatives were particularly suitable for use in DDS. Choi et al. (2012) designed a drug release system activated by UVA irradiation using 2*-*nitrobenzyl as the photo-cleavable linker for the light-mediated release of methotrexate. They also used 2*-*nitrobenzyl linkers to extend the release of methotrexate from a fifth-generation poly(amidoamine) dendrimer carrier. Hu and colleagues (Hu et al., 2017) developed photo- and chemo- cleavable compounds by combining a 2*-*nitrobenzyl ester moiety as a photo-cleavable linker within multi-block polystyrenes. These polymers could be cleaved not only using UVA light but also by ester group hydrolysis.

**Figure 4:**
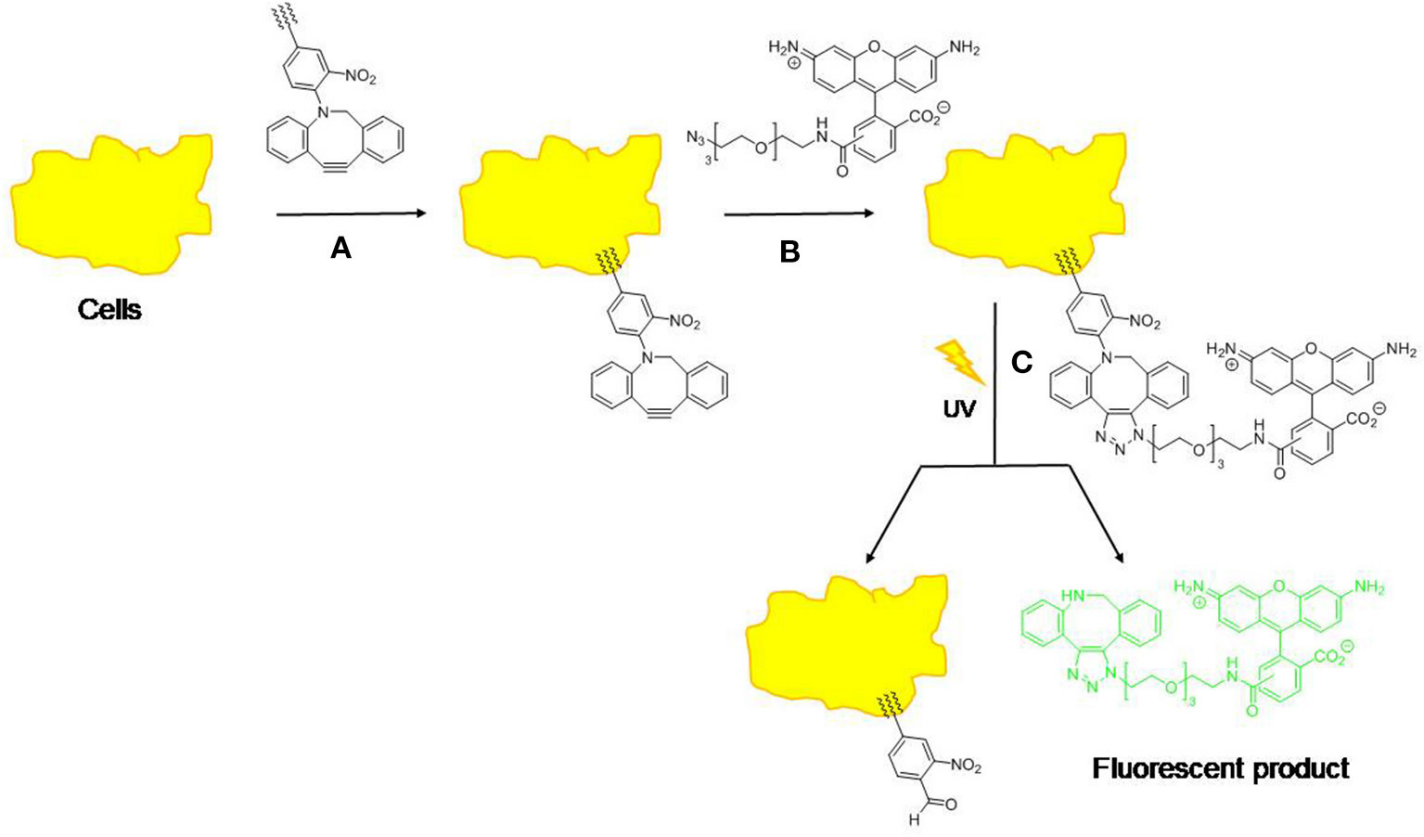
Approach to the analysis of cell membrane delivery and release using copper-free “click” chemistry and a photo-cleavage. (1) Cells are treated with lipid-tethered compound; (2) Cells are treated with “azide fluor 448” to give ‘clicked' compound; (3) Cells are treated with UV light to effect photo-cleavage and release fluorophore (λ_*em*_ = 501 nm, λ_*ex*_ = 525 nm).

Another commonly used linker for photo-cleavage linker is coumarin and its derivatives, since faster release rates are achieved compared to 2*-*nitrobenzyl (Beauté et al., 2019). Coumarin derivatives possess longer absorption wavelengths, larger molar extinction coefficients, higher stability, and biocompatibility, and strong fluorescent properties (Gao et al., 2017). Jiang et al. (2017) investigated three different coumarin dimer isomers using time-resolved transient absorption spectroscopy in order to verify the mechanism of photo-cleavage. This revealed that the cleavage mechanism was *via* a non-fluorescent, short-lived (<200 fs) singlet reaction state. Chung et al. (2013) also analyzed the effect of coumarin on polymer model stating that the increase in coumarin functionality resulted in decreasing nanoparticle size and polydispersity and increased the stability of the polymer in water. Hence, Soares et al. (2017) examined light-sensitive moieties for releasing bioactive molecules by combining coumarin with oxazoles using butyric acid resulting in a shorter irradiation time at a longer wavelength (11 min, at 350 nm), thus demonstrating coumarin can enhance the photo-cleavage of the prodrug. A combination of coumarin with (6-bromo-7-methoxycoumarin)-nicotinamide reported by Bourbon et al. (2013) also showed photo-cleavage upon irradiation. Nazemi and Gillies (2014) reported the synthesis of polyester dendrimers incorporating 2*-*nitrobenzyl photo-cleavable linkers. Complete photolytic cleavage of the dendrimer backbone without formation of macromolecular byproducts was observed upon illumination with UV light. Liu et al. (2019) reported a low-molecular-weight hydrogelator (LMWG) that contained coumarin derivatives. The hydrogel underwent photo-cleavage at the C-N bond in 7-aminocoumarin upon irradiation at 365 nm. Alam et al. (2015) also modified the liposomes to enable photo-induced cleavage by synthesized photo-cleavable lipid analogs with 2-nitrobenzyl moiety close to the head-group of the lipid backbone to maximize bio-conjunction efficiency and photo-release (Figure 4). Delivery of the liposome containing the photo-cleavable lipid to live cells was also demonstrated. Another study by Radl et al. (2015) combined an epoxy-based polymer with a 2*-*nitrobenzyl ester (*o-*NBE). They successfully demonstrated the efficiency of photo-cleavage upon UV radiation and this technology could be applied to thin composite materials.

### Photo-Crosslinking

Photo-crosslinking occurs through irradiation of polymerizable double bond either directly or by use of a sensitizer or radical initiator with consequent release of molecules (Fomina et al., 2012; Tunc et al., 2014). This mechanism has been used in drug delivery systems utilizing nanoparticles and stabilizing therapeutic nanocarriers such as nano-capsules and liposomes (Barhoumi et al., 2015). Photochemical crosslinking of polymers occurs upon UV irradiation at 364 nm, and de-crosslinking at 254 nm, affecting a decrease in nanoparticles size for cargo release from nanocarriers (Karimi et al., 2017). Photo-crosslinking is usually used in the formation of nanoparticles but it can be utilized in photo-triggered drug release (Fomina et al., 2012).

Some photo-crosslinking materials need radical initiators to perform crosslinking under UV irradiation. For example, Chesterman et al. (2018) compared cinnamoyl groups and coumarin within functionalized copolymers. The coumarin functionalized aliphatic polycarbonates demonstrating effective crosslinking upon UVA irradiation at wavelengths between 320 and 400 nm with the maximum intensity being 365 nm. Ding et al. (2011) analyzed some copolymers incorporating the cinnamyloxy group by synthesizing poly(ethylene glycol monomethyl ether)-β-poly(*L*-glutamic acid-co-γ-cinnamyl-*L*-glutamate) [mPEG-β-P(LGA/CLG)] and poly(*L*-glutamic acid-co-γ-cinnamyl-*L*-glutamate)-β-poly(ethylene glycol)-β-poly(*L*-glutamic acid-co-γ-cinnamyl-*L*-glutamate) (P(LGA/CLG)-β-PEG-β-P(LGA/CLG)) by using ring-opening polymerization (ROP) of γ-benzyl-*L*-glutamate-N-carboxy anhydride (BLG-NCA) monomer with a PEG-based macro-initiator. The results revealed the micelle polymer [P(LGA/CLG)] core block was able to crosslink under UVC irradiation at 254 nm wavelength due to cinnamyloxy photo-dimerization. Similarly, Teixeira et al. (2013) used benzophenone to induce photo-crosslinking during their synthesis of water-based poly(ethylene oxide) (PEO) polymers and successfully demonstrated photo-crosslinking upon UV radiation.

Liposomes are an alternative drug delivery system when using photo-induced crosslinking of lipids to enhance drug release efficiency. This results from the liposome lipid bilayer undergoing crosslinking upon UV irradiation (Leung and Romanowski, 2012). This mechanism is illustrated by research conducted by Yavlovich et al. (2009), in which they designed a light-triggered liposome from photo-polymerizable phospholipid DC-8,9-PC (1,2-bis(tricosa-10,12-diynoyl)-sn-glycero-3-phosphocholine) and 1,2-dipalmitoyl-sn-glycero-3-phosphocholine, which was used to encapsulate the fluorescent dye calcein. Exposure of this liposome to UV irradiation at 254 nm resulted in DC-8,9-PC cross-linking and release of the fluorescent dye. This finding proved that UV light-induced crosslinking of the liposome lipid bilayer resulted in release of its contents (Yavlovich et al., 2009). Photo-crosslinking of the liposome resulting in drug release occurred not only at 254 nm but also at 514 nm. At 254 nm, the photo-crosslinking resulted in liposome membrane disruption and polymerization, while at 514 nm the mechanism was related to ROS production (Miranda and Lovell, 2016). Liposomes containing DC-8,9-PC loaded with doxorubicin have been used to demonstrate their potential *in vitro* and in cell-based assays (Puri, 2014). Significantly, irradiation at 514 nm resulted in increased cytotoxicity compared to irradiation at 254 nm. Although the exact mechanism for doxorubicin release was not determined (Miranda and Lovell, 2016), it appears to be related to a type I photo-reaction involving electron transfer and generations of ${\text{O}}_{2}^{-}$ and H_2_O_2_ (Yavlovich et al., 2013).

### Photo-Induced Rearrangement

Exposure to light causes Wolff rearrangement of some molecules, and this has been used to trigger drug release from micelles either using NIR at 800 nm or UV radiation at 350 or 365 nm (Olejniczak et al., 2015). For a Wolff rearrangement to occur, α-diazocarbonyl compounds eliminate nitrogen and form a ketene intermediate, which subsequently reacts with nucleophiles such as water, alcohols, or amines to form the corresponding carboxylic acids, esters or amides, respectively. Ketenes can also undergo [2+2] cycloaddition to form compounds with four-membered rings (Cui and Thiel, 2013; Barhoumi et al., 2015).

Liu et al. (2012) conducted research using a dextran-graft-(2-diazo-1,2-naphthoquinone) (Dex-DNQ) amphiphilic copolymer in which the hydrophilic dextran moiety was modified with the hydrophobic DNQ. The DNQ undergoes Wolff rearrangement upon UV irradiation, triggering drug release from Dex-DNQ micelles. Similarly, Sun et al. (2014) designed light-responsive linear-dendritic amphiphiles (PEO-D3DNQ) complexed with DNQ. The DNQ absorption was reduced upon irradiation at 365 nm, suggesting that the Wolff rearrangement had occurred. Upon UV irradiation, the PEO-D3DNQ polymer was disrupted releasing the encapsulated drug *via* conversion of the hydrophobic DNQ into hydrophilic indene-3-carboxylic acid. Recently, Li et al. (2018) combined UV/NIR light and pH to effect controlled release using amphiphilic polymers of poly(ethyleneglycol)-block-poly(dimethylaminoethyl methacrylate) modified with DNQ (PEG-PDMAEMA-DNQ). Upon irradiation with UVA (365 nm), DNQ was converted into indene-3-carboxylic acid to release coumarin from micelles (Chesterman et al., 2018). This study reveals that the combination of UV light and pH-responsiveness can be used to trigger drug release.

## Application of UVA-triggered Drug Release

UV light is able to stimulate drug release through mechanisms such as photo-isomerization, photo-cleavage, photo-crosslinking, and photo-induced rearrangement. This typically triggers a conformational change in the DDS, releasing the drug (Barhoumi et al., 2015). Compared to other light-triggers, short-wavelength UV light possesses higher energy which is able to break down molecular structures such as covalent bonds or promotes isomerization between different conformations, and achieves a much better response (Yang et al., 2017). Herein, the application of UVA drug release as a strategy for photo-therapy, photo-protection, and caged iron chelators is discussed for use in smart sunscreens and other DDS.

### Photo-Therapy

Considering the utility of UVA-triggered drug release, many studies are focusing on combining light-triggered release to enhance the advantages of photo-therapy especially in the use of photodynamic therapy (PDT) or photo-thermal therapy (PTT) (Cao et al., 2018). Since UCNPs have been established to exploit UV, many studies employ the combination of UCNPs with photo-therapy (Qiu et al., 2018). UCNPs provide the strategy of up-conversion to utilize the low toxicity and deep penetration of NIR (Silva et al., 2019) to overcome the limitations of photo-toxicity and low depth tissue penetration (around 10 mm) (Mura et al., 2013) by UV light. The two or multiple photons with low energy from NIR light are converted into high energy of one emitted photon in UV light (Zhong et al., 2014; Jafari and Rezvanpour, 2019). Thus, the approach appears to overcome the limitations of using UV in photo-therapy and photo-drug release (Marturano et al., 2019). The application of UCNPs also offers the possibility of using NIR irradiation energy to elicit photo-chemistry which usually requires UV or visible light. Hence some UCNPs contain Yb^3+^ and Tm^3+^ as a converter to emit UV light upon NIR excitation at 980 nm to improve their ability to release drug (Wang et al., 2013). Since NIR light provides deep tissue penetration, the conversion of NIR to UV light allows more effective and efficient drug release from UCNPs, whilst minimizing tissue damage and cell death (Lee and Park, 2018) by reducing light scattering within the biological sample (Raza et al., 2019).

**Figure 5:**
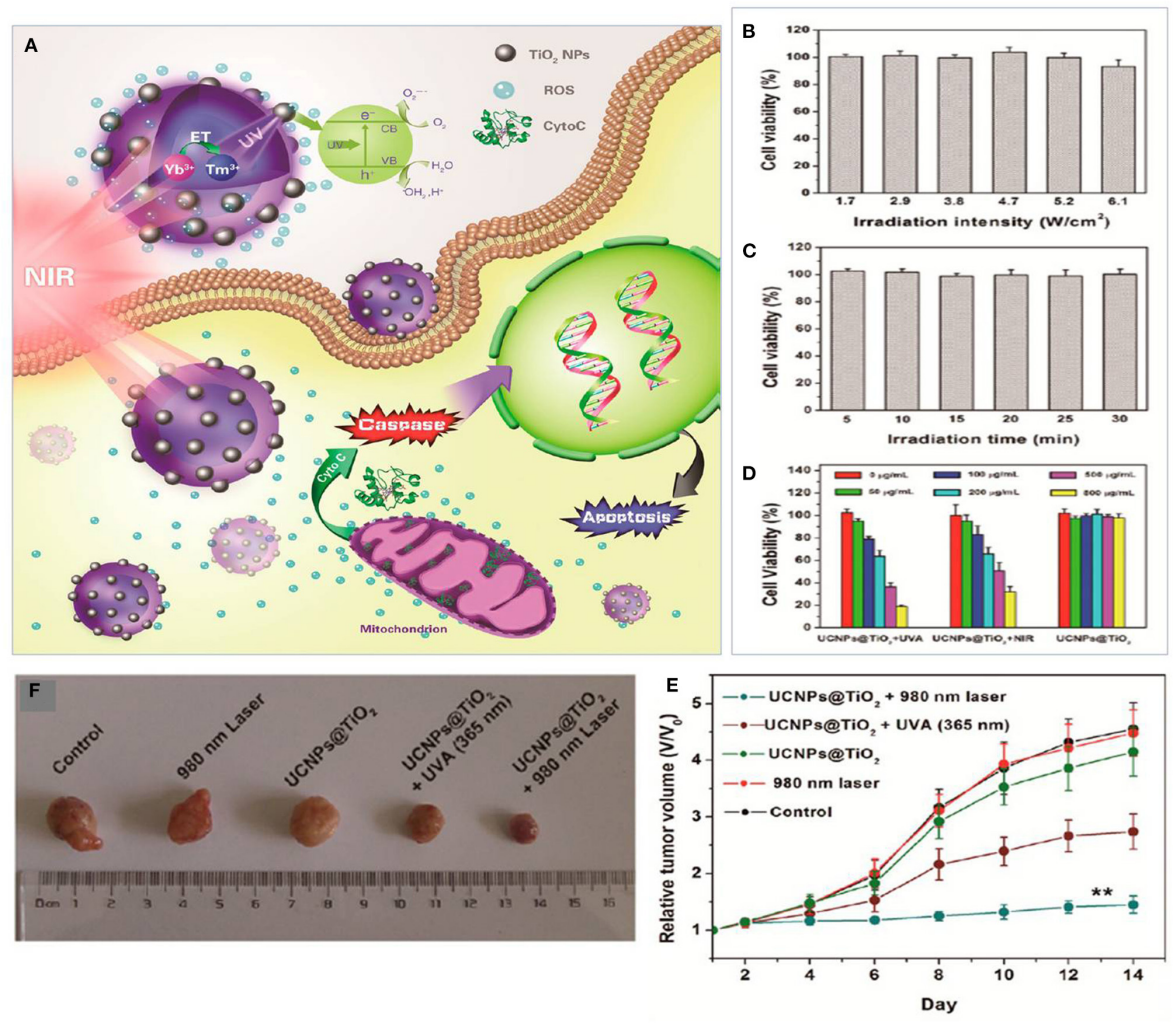
**(A)** Plot for the potential molecular mechanism of inducing apoptosis with UCNPs@TiO2-based NIR light-mediated PDT treatment. **(B)** Cell viability after 980 nm laser irradiation for different intensities for 30 min (5 min break after 10 min of irradiation). **(C)** After UV light irradiation at 365 nm for different irradiation times. **(D)** *In vitro* viability of HeLa cells treated with UCNPs@TiO2 NCs. **(E)** *In vitro* tumor volume changes of tumor-bearing mice in different groups after various treatments. **(F)** Digital photograph of excised tumors from representative mice after various treatments. Data for **(B–E)** are means ± SD, n = 3. Adapted with permission from Hou et al. (2015). Copyright 2015, American Chemical Society.

Hou et al. (2015) revealed that the combination of UCNPs@TiO_2_-based NIR light-mediated PDT triggers cancer cell death through the mitochondria-apoptosis pathway. As reported, their UCNPs had a TiO_2_ shell with a NaYF4:Yb^3+^core (see Figure 5). The use of Tm^3+^@NaGdF4:Yb^3+^ enhanced the up-conversion of NIR into UV upon irradiation of TiO_2_ (photosensitizer) to form extracellular and intracellular ROS which in turn induced tumor inhibition. Similarly, Hou et al. (2017) introduced UCNPs@mSiO_2_-(azo+RB) (azobenzene + Rose Bengal) nano-impellers which utilized the UCNP core to release doxorubicin (DOX) and generate ROS in a combination of chemotherapy and PDT. Based on their study, NIR light at 980 nm was used to produce UV light within the UCNPs core-shell, which activated azobenzene with consequent release of doxorubicin and activation of Rose Bengal as a photosensitizer for the generation of cytotoxic ROS to maximize the treatment.

Recently, Bi et al. (2018) investigated photo-therapy treatment by combining PDT and PTT utilizing UCNPs composed of graphene oxide and ZnFe_2_O_4_. They used Tm^3+^ at the core of the UCNPs to convert NIR into UV emission, which activated graphene oxide and ZnFe_2_O_4_to achieve PTT. Moreover, this strategy triggered ZnFe_2_O_4_ to generate ROS and other toxic radicals by reaction with H_2_O_2_ in the tumor microenvironment. This strategy was able to overcome one of the limitations of drug release using UV light, since these UCNPs had reduced cytotoxicity to normal cells and highly cytotoxic to cancer cells.

Huang et al. (2017) focused on the utilization of nanoscale ZnO because of its potential as a smart drug delivery system due to its biocompatibility, ease of modification, and good properties and structures as well as low cost. A previous study on ZnO and benzophenone-3 (Huang et al., 2013) demonstrated the cyclic hydrophobic to hydrophilic switching and *vice versa* under UV and dark exposure. Building on this, Huang et al. (2015) developed nanocomposites of Fe_3_O_4_@ZnO core-shell to load docetaxel and encapsulate the epidermal growth factor (EGF) on the polymer. Upon stimulation with UV irradiation, the chemical structure of ZnO was rearranged to release docetaxel and EGF, resulting in obstruction of the growth of cancer cells (Huang et al., 2015). In further research, they designed a photo-responsive multifunctional drug delivery by combining Fe_3_O_4_@ZnO core-shell and amphipathic polymer of P(BA-co-HBA)-loaded docetaxel which was able to boost chemotherapy effects by UV stimulation to trigger the ZnO hydrophilic / hydrophobic transition to release the drug and to prompt the amphipathic polymer to adsorb and encapsulate EGF (Huang et al., 2016). Later, Kong et al. (2017) used the same multifunctional DDS to decrease the growth of skin cancer by giving a low dose of UV to release docetaxel and absorb EGF. A study from Huang et al. (2016) also demonstrated that UV-mediated drug release would be able to control docetaxol release with simultaneous EGF adsorption resulting in reduced proliferation and metastasis of cancer cells.

### Photo-Protection

**Figure 6:**
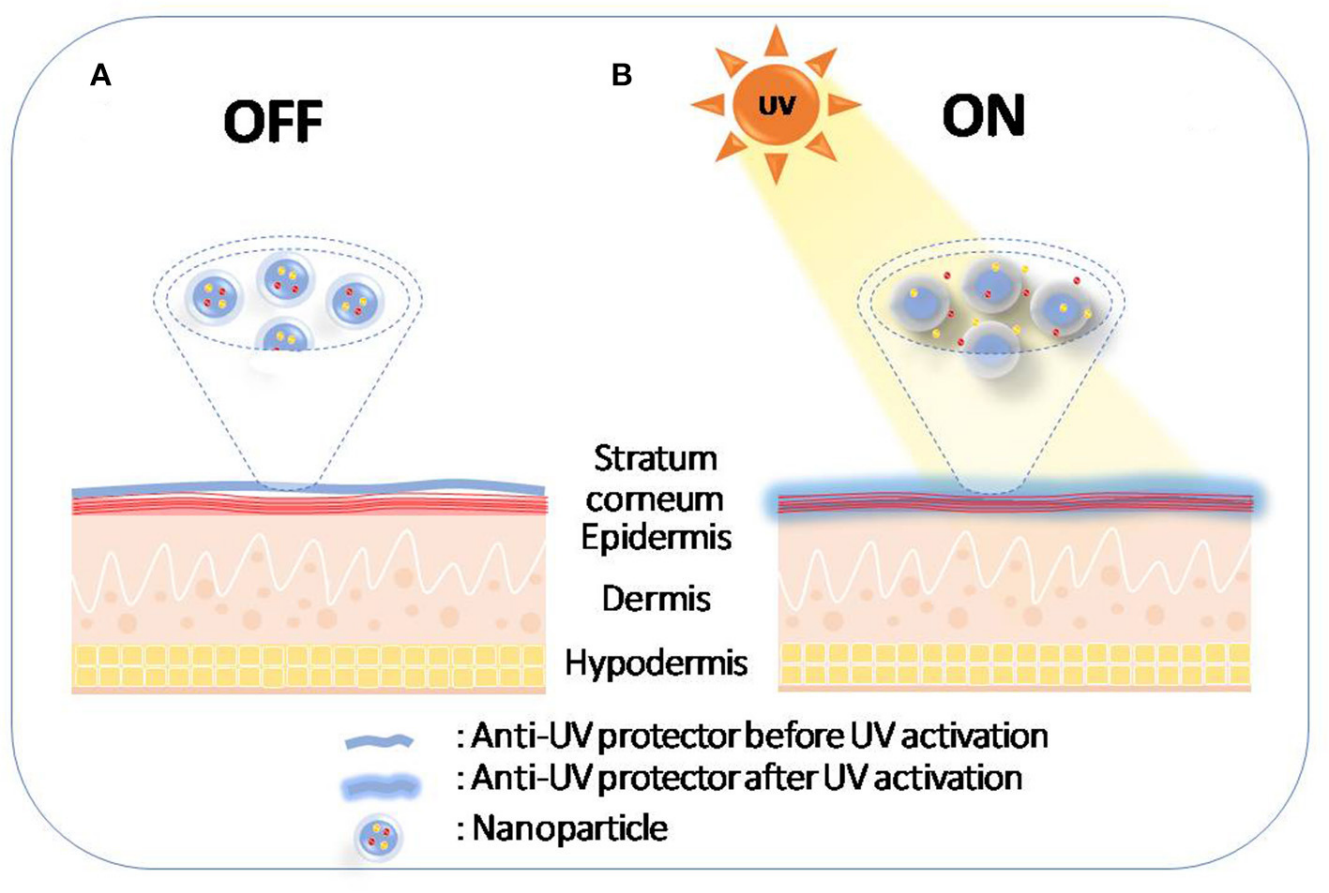
Schematic diagram of photo-protection by UVA-induced drug release on the skin. **(A)** Nanoparticles remain on stratum corneum before sun exposure; **(B)** Nanoparticles release the drug after sun exposure.

UV radiation contributes to 65% of melanoma cases and almost 90% of non-melanoma skin cancers and has gained much attention as it triggers other hazardous effects including acute and chronic skin damage, photo-aging, and immune system effects (Amaro-Ortiz et al., 2014; Kim and He, 2014; Gilbertz et al., 2018). It is becoming essential to protect body parts from exposure to UV radiation as a preventive strategy using sunscreens for photo-protection (Yeager and Lim, 2019), which contain UV filters to reflect, absorb, and disperse the energy of solar illumination (Geoffrey et al., 2019). A new sophisticated technology allows the utilization of the drug delivery systems in skin protection. Since human skin is an exposed surface, external stimuli such as heat, visible light or UV light can be used to control drug release on the skin for protection and to treat inflammation (Vogt et al., 2016). This idea offers the possibility of activating drug release upon UV exposure to protect the skin when it is most needed (Geoffrey et al., 2019) (Figure 6).

Previously, Huang et al. (2015) studied a smart drug release system for skin protection by using ZnO nanoparticles as a UV filter loaded with benzophenone-3. The system was tested on keratinocytes and skin fibroblasts to investigate drug release upon UV irradiation. The ZnO NPs were able to interconvert between hydrophobic and hydrophilic states upon light and dark exposure, respectively. Almost all benzophenone-3 was released after 2 h of UV exposure (~100 kJ/m^2^) with light switch on and off each 30 min, and low cytotoxicity for the nanoparticles was observed for human keratinocytes and fibroblasts. Their findings demonstrated the UV-triggered drug release as a skin protection strategy which initiated further studies. In a follow-up study (Huang et al., 2020), the same group investigated acetyl-11-keto-β-boswellic acid (AKBA)-loaded ZnO nanoparticles for UVA (160 kJ/m^2^) protection. AKBA possesses outstanding anti-inflammatory and antioxidant effects, and the nanoparticles had low cytotoxicity to HaCaT cells.

Similarly, Aparici-Espert et al. (2018) introduced photo-caged sunscreens for UV responsive release by combining the nonsteroidal anti-inflammatory drug diclofenac with avobenzone as a solar filter utilizing a phenacylavobenzone pro-drug. Photolysis of the dyad in a hydrogen-donor solvent resulted in simultaneous release of diclofenac and avobenzone. Another study from Suh et al. (2019) formulated a smart sunscreen by encapsulating padimate O UVB filters within biodegradable nanoparticles BNPs consisted of poly(_D,L_-lactic acid)-hyperbranched polyglycerol containing avobenzone and octocrylene. This study found that the incorporation of a UV filter within nanoparticles offered filter stabilization and optimized drug release upon UV irradiation. The result also showed water-resistance of formulated sunscreen to be around 85% particle retention after 3 h. The results indicated biodegradable nanoparticles were able to protect and release the drug upon UV illumination to protect the skin.

### Caged Iron Chelators

Irradiation of human skin cells with physiological doses (up to 250 kJ/m^2^) of UVA also result in release of labile iron from stores with consequent generation of ROS that contribute to skin photo-aging and photo-carcinogenesis (Bissett et al., 1991; Pourzand et al., 1999). The increased levels of ROS foster oxidative stress in subcellular compartments notably lysosomes, mitochondria, plasma membranes, and even nuclear membranes (Reelfs et al., 2004; Aroun et al., 2012). In skin fibroblasts, UVA-induced damage to mitochondrial membranes leads to immediate ATP depletion which in conjunction with damage to plasma membranes promote necrotic cell death (Zhong et al., 2004; Aroun et al., 2012). It is necessary to protect the skin cells from iron overload by regulating and restricting the absorption of iron as well as regulating iron-binding to proteins such as ferritin, transferrin, and hemoglobin (Powers and Buchanan, 2019; Katsarou and Pantopoulos, 2020). However, upon UVA radiation, the iron homeostasis is severely compromised in skin cells due to proteolytic degradation of the iron storage protein ferritin which contributes to accumulation of potentially damaging intracellular labile iron observed immediately after UVA irradiation. Therefore, iron chelators can be used to remove the UVA-mediated excess labile iron within the cell and consequently suppress the production of higher amounts of ROS via Fenton chemistry (Jomova and Valko, 2011; Cabantchik, 2014; Abbate and Hider, 2017; Koppenol and Hider, 2019). However, iron chelators need to be used carefully in order to remove excess iron without disrupting the essential iron homeostasis within the cells (Reelfs et al., 2010; Powers and Buchanan, 2019; Zhou et al., 2019).

To avert excessive depletion of iron by chelators, it is imperative to design a smart chelating agent as a prodrug which is released only upon UV irradiation of the skin (Reelfs et al., 2010). As mentioned previously, our group (Yiakouvaki et al., 2006) introduced a series of light-activated iron chelators salicylaldehyde isonicotinoyl hydrazone (SIH) and pyridoxal isonicotinoylhydrazone (PIH) which were caged using the 1-(2-nitrophenyl)ethyl group. Under normal/dark conditions (without UVA exposure), the caged iron chelator was stable and did not deplete the labile iron pool within the cell. In contrast, upon exposure to UVA radiation the chelator was released, which protected the skin cells and prevented the UVA-induced necrosis. These results support the application of UVA-induced drug release as a future skin photoprotection strategy.

Pelle et al. (2011) extended the previous study by examining (2-nitrophenyl)ethyl pyridoxal isonicotinoyl hydrazone (2-PNE-PIH) as an iron chelator pro-drug that was activated upon UVA irradiation whilst minimizing the effect on essential iron metabolism. The prodrug was activated by UVA irradiation to release the active chelator PIH which sequestered the excess labile iron which was concomitantly produced by UVA in keratinocytes. This provoked a dose- and context-dependent protection against UVA damage in keratinocytes. The light-activated iron chelators have therefore a great potential as powerful sunscreen ingredients with superior effectiveness than antioxidants. The antioxidants only neutralize one cycle of ROS formation without affecting the harmful labile iron that will continue to catalyze the formation of harmful ROS (Wang et al., 2014). Franks et al. (2015) also synthesized a multifunctional caged compound PC-HAPI [2-((E)-1-(2-isonicotinoyl hydrazono) ethyl) phenyl *trans*-3-(2,4-dihydroxyphenyl) acrylate] with a caging moiety based on trans-(*o*-hydroxy) cinnamate ester which was photocleavable upon UVA exposure to release a coumarin-based natural antioxidant umbelliferone, and an aroylhydrazone metal chelator, HAPI [N′-[1-(2-hydroxyphenyl)ethyliden] isonicotinoylhydrazide]. The latter light-activatable compound was used to tackle the dual damaging effects of UVA component of sunlight by neutralizing the ROS and harmful labile iron released by the radiation in the skin cells (Pourzand and Tyrrell, 1999; Pourzand et al., 1999). Since unprotected iron-chelators represent a risk of toxicity due to non-targeted iron depletion, multifunctional pro-chelators that have little or no affinity for iron until activated by external stimuli (e.g., ROS) have gained ground as cardio-protective and neuroprotective agents (Jansová and Šimunek, 2019; Wu et al., 2020).

**Figure 7:**
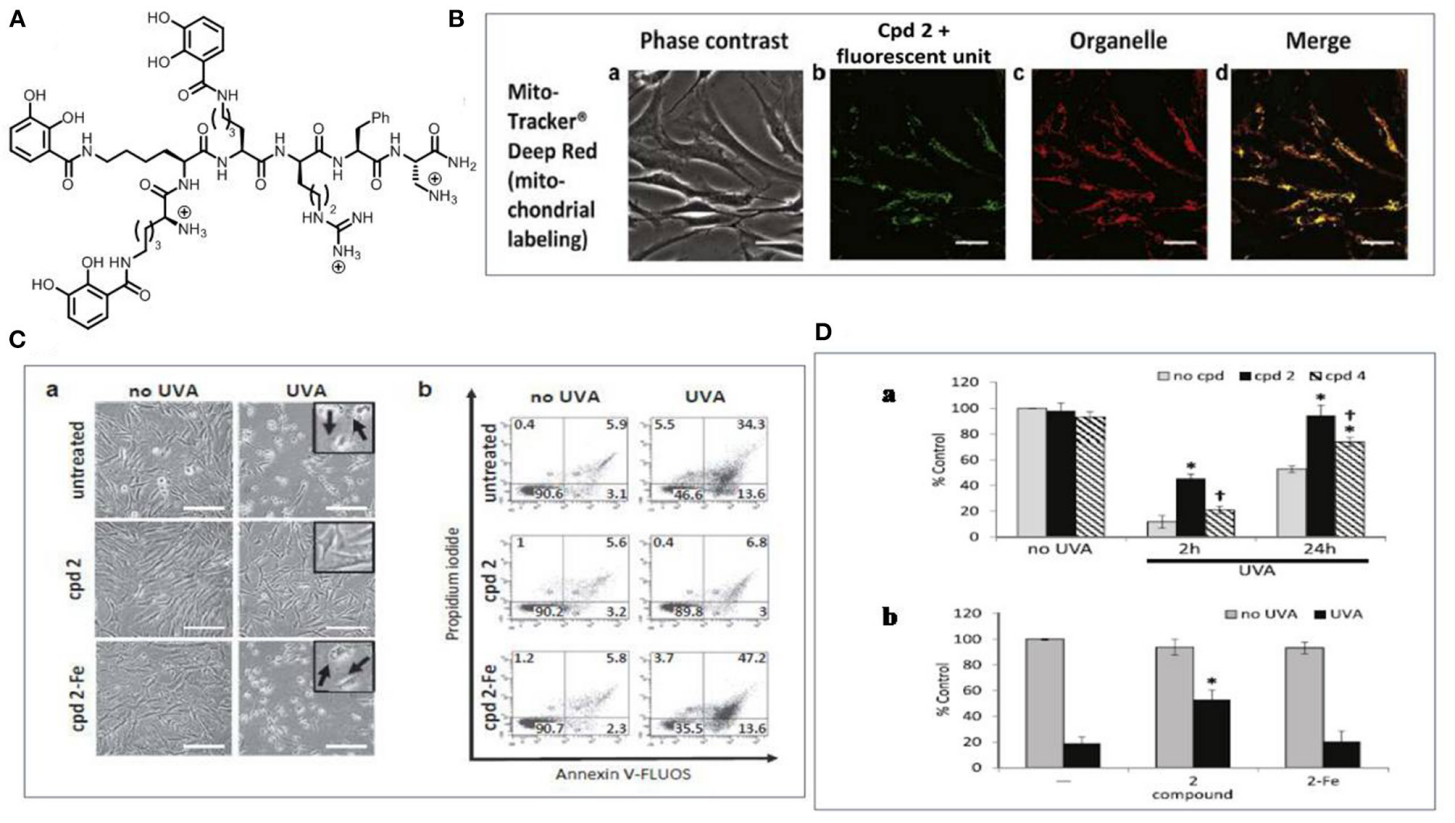
The use of mitochondrial targeted tricatechol iron chelator for skin photoprotection. **(A)** The structure of the chelator (compound 2). **(B)** Microscopy analysis of the subcellular localization of compound 2 tagged with a fluorescent unit (a) Phase contrast; (b) Compound 2 with the fluorescent unit; (c) Mitochondria stained with mito tracker (d) Merged data. Scale bar = 10 μm; **(C)** Compound 2 protects FEK4 cells from UVA-induced cell death (a) Bright-field images were captured 24 h after treatment. Swelling (arrow in insert) is indicative of cell death by necrosis and is visible after UVA treatment alone or in the combination of compound 2-Fe. Scale bar = 50 μm; (b) Cells analyzed by flow cytometry. Live cells are defined as Annexin V-negative/PI-negative (lower left-hand quadrant). **(D)** Compound 2 significantly reduces UVA-induced damage to mitochondria membrane (a) Bar chart of the results of TMRM staining experiment; (b) FEK4 cells were pre-treated with either compound 2 alone or as a complex with iron. Data are means ± SD, *n* = 3-5. TMRM, tetramethylrhodamine methyl ester; UVA, ultraviolet A. Adapted with permission under the terms of CC BY 4.0 license (Reelfs et al., 2016). Copyright 2016, The Authors. Published by Elsevier.

Another challenge in using iron chelators for skin photoprotection is to target the chelating agent to the heart of oxidative damage. The mitochondrial-targeted iron chelators are reported (Reelfs et al., 2016) to provide unprecedented protection against UVA-induced damage in skin fibroblasts. The design of mitochondria-targeted chelators was based on the observation that mitochondria were a significant source of ROS whilst maintaining iron homeostasis (Levi and Rovida, 2009; Aroun et al., 2012). We designed a mitochondria-targeted hexadentate iron chelator based on the tricatechol motif (compound 2) (Figure 7). Upon UVA irradiation, the mitochondria-targeted iron chelator protected the skin cells from damage by reducing the organelles' labile iron levels and consequent ROS levels. The above studies support the development of UVA-induced caged iron chelators to protect the skin from the detrimental effect of sunlight as a promising strategy for skin protection.

## Conclusions and Perspectives

The introduction of light-controlled drug delivery systems has greatly improved the targeted delivery since the use of an external stimulus such as light gives many benefits. The intrinsic properties of light mean that spatially and temporally controlled release in a wide range of therapeutic applications can be easily achieved. Hence, it provides the possibility of controlling the power of the stimulus and the ability to focus it onto a specific area and penetrate into body tissues by choosing an appropriate wavelength of light (Barhoumi and Kohane, 2015; Bagheri et al., 2016; Liu et al., 2016; Shim et al., 2017).

UVA in the range of 320–400 nm has been used in some treatments of skin conditions and also in DDS. Despite the photo-toxicity of UVA resulting in damage to the cell, UVA shows significant potential as a stimulus for drug release. The higher energy of the UVA light means that it is possible to breakdown covalent bonds within molecular structures and hence some materials are highly responsive to UV light (Breuckmann et al., 2004; Svobodova et al., 2006; Jin et al., 2011). In order to achieve optimum usage of UVA for photo-triggered drug delivery, mechanisms such as photo-isomerization, photo-cleavage, photo-crosslinking, and photo-induced rearrangement can be utilized. These mechanisms induce changes in covalent bonding of the material or other conformational changes.

More recently, some applications in clinical photo-therapy such as PDT and PTT have been established to capitalize on the benefits of the controlled-release drugs by UV. Some systems also utilize up-conversion to improve therapeutic outcomes by converting NIR to UV light since NIR light can penetrate deeply into tissues and is much less harmful than UV light (Qiu et al., 2018; Jafari and Rezvanpour, 2019; Marturano et al., 2019). It is also possible to utilize UVA-induced drug release to release a photo-protective agent upon exposure to sunlight (Vogt et al., 2016; Geoffrey et al., 2019; Yeager and Lim, 2019). Since UVA-induced drug release is only activated upon UV irradiation, the use of these pro-drugs within sunscreens offer a promising approach for skin protection during the exposure to daily sunlight. The discovery of caged iron chelators is a more recent development in the area of UVA-activated drug release in smart sunscreens, minimizing damage by preventing ROS formation whilst also minimizing non-specific iron depletion (Basu-Modak et al., 2006; Yiakouvaki et al., 2006; Levi and Rovida, 2009; Reelfs et al., 2016).

Taking all of these aspects into consideration, UVA-induced drug release shows great promise as a means of controlling drug release with many potential applications. These include the use of UV radiation for controlled drug release and the use of smart sunscreens to protect the skin cells against sunlight-mediated UV damage.

## Author Contributions

VK, WW, CP, and JZ contributed on conceptualized of the study. VK and MN wrote the manuscript, ML, HL, MDL, and JZ assisted in writing of the manuscript. WW, ML, HL, MDL, CP, and JZ contributed to the revision of the manuscript for important intellectual content. All authors contributed to the writing of this manuscript and approved the final version to be published and agree to be accountable for all aspects of this work.

## Conflict of Interest

The authors declare that the research was conducted in the absence of any commercial or financial relationships that could be construed as a potential conflict of interest.
